# The cumulative therapeutic effect of acupuncture in patients with migraine without aura: Evidence from dynamic alterations of intrinsic brain activity and effective connectivity

**DOI:** 10.3389/fnins.2022.925698

**Published:** 2022-07-19

**Authors:** Yilei Chen, Yingjie Kang, Shilei Luo, Shanshan Liu, Bo Wang, Zhigang Gong, Yanwen Huang, Hui Wang, Songhua Zhan, Wenli Tan

**Affiliations:** ^1^Department of Radiology, Shuguang Hospital Affiliated to Shanghai University of Traditional Chinese Medicine, Shanghai, China; ^2^Department of Acupuncture and Moxibustion, Shuguang Hospital Affiliated to Shanghai University of Traditional Chinese Medicine, Shanghai, China

**Keywords:** migraine, Granger causality analysis, amplitude of low-frequency fluctuations, dynamic analysis, acupuncture

## Abstract

We explored the dynamic alterations of intrinsic brain activity and effective connectivity after acupuncture treatment to investigate the underlying neurological mechanism of acupuncture treatment in patients with migraine without aura (MwoA). The Functional Magnetic Resonance Imaging (fMRI) scans were separately obtained at baseline, after the first and 12th acupuncture sessions in 40 patients with MwoA. Compared with the healthy controls (HCs), patients with MwoA mostly showed a decreased dynamic amplitude of low-frequency fluctuation (dALFF) variability in the rostral ventromedial medulla (RVM), superior lobe of left cerebellum (Cerebellum_Crus1_L), right precuneus (PCUN.R), and so on. The decreased dALFF variability of RVM, Cerebellum_Crus1_L, and PCUN.R progressively recovered after the first and 12th acupuncture treatment sessions as compared to the baseline. There was gradually increased dynamic effective connectivity (DEC) variability in RVM outflow to the right middle frontal gyrus, left insula, right precentral gyrus, and right supramarginal gyrus, and gradually enhanced DEC variability from the right fusiform gyrus inflow to RVM. Furthermore, the gradually increased DEC variability was found from Cerebellum_Crus1_L outflow to the left middle occipital gyrus and the left precentral gyrus, from PCUN.R outflow to the right thalamus. These dALFF variabilities were positively correlated with the frequency of migraine attacks and negatively correlated with disease duration at baseline. The dynamic Granger causality analysis (GCA) coefficients of this DEC variability were positively correlated with Migraine-Specific Quality of Life Questionnaire scores and negatively correlated with the frequency of migraine attacks and visual analog scale (VAS) scores after 12th acupuncture sessions. Our results were analyzed by a longitudinal fMRI in the absence of a sham acupuncture control group and provided insight into the dynamic alterations of brain activity and effective connectivity in patients with MwoA after acupuncture intervention. Acupuncture might relieve MwoA by increasing the effective connectivity of RVM, Cerebellum_Crus1_L, and PCUN.R to make up for the decreased dALFF variability in these brain areas.

## Introduction

Migraine is a prevalent primary headache disorder characterized by recurrent headache attacks, nausea or vomiting, and being sensitive to sound or light (Olesen et al., 2018). Migraine is a disabling and common neurological disorder with a 1-year prevalence of 12% in the general population (Schwedt et al., 2015), and prevalence in China is 9.3%, with a female to male ratio of about 2:1 (Yu et al., 2012). Migraine without aura (MwoA) is the most common type of migraine. Patients with migraine usually have frequent, severe, and disabling headache attacks, which cause an enormous individual and social burden (Leonardi et al., 2005). Migraine is typically treated by various pharmacological or non-pharmacological therapies to relieve pain or reduce migraine attacks. However, these methods may have limited efficacy and special patient populations. And some of them have shown adverse effects, such as weight gain, fatigue, sleep disturbance, gastrointestinal reaction, or medication overuse (Diener et al., 2015). Acupuncture, as one of the treatment modalities of Traditional Chinese Medicine (TCM), is widely used as a complementary and alternative treatment to prevent migraine attacks and relieve pain during a migraine in China. Acupuncture was recognized to migraine for its long-term prophylactic effect (Zhao et al., 2017; Xu et al., 2020). However, the mechanism of the cumulative impact of acupuncture treatment is currently unclear.

Recently, neuroimaging has provided new insight into understanding the central mechanism of acupuncture on migraine (Chang et al., 2021; Liu L. et al., 2021). Several Functional Magnetic Resonance Imaging (fMRI) studies have indicated that verum and sham acupuncture have different modulation effects on the amplitude of low-frequency fluctuation (ALFF) of the rostral ventromedial medulla (RVM)/trigeminocervical complex (TCC) in patients with migraine (Li et al., 2017b). Verum acupuncture elicited a more widespread and remarkable cerebral response, including the pain matrix, lateral and medial pain system, default mode network, and cognitive components of the pain processing system, compared to sham acupuncture (Zhao et al., 2014). Verum acupuncture could also normalize the abnormal network connectivity in the visual, default mode (DMN), sensorimotor, and frontal-parietal networks (Tu et al., 2020). Furthermore, it was demonstrated that acupuncture treatment could increase the functional connectivity (FC) of the right frontoparietal network (Li et al., 2017a). The connectivity of DMN was normalized after acupuncture intervention (Zou et al., 2019). Our previous studies demonstrated that acupuncture could improve the dysfunction of the cerebellum and activate the brain regions involved in the modulation of pain and emotion in patients with MwoA by the regional homogeneity (ReHo) analysis method (Liu S. et al., 2021). However, most of these studies above have focused on the static characterizations or traditional unidirectional FC of the brain. They have not explored the continuous effects of different periods of acupuncture. Brain activity is inherently dynamic (Bassett and Sporns, 2017). Recently, a number of studies have proposed that brain activity was dynamically changed over time, so dynamic ALFF was an effective tool to explore dynamic brain activity in healthy people (Liao et al., 2019). The dynamic ALFF method measured the variance of ALFF over time combining it with “sliding-window” approaches (Cui et al., 2020; Zhao et al., 2021). A tapered window, created by convolving a rectangle with a Gaussian, was used for segmenting extract time courses (Fu et al., 2021). The time-varying brain activity characterized by dynamic ALFF may underline the disruption of brain activity in various mental disorders (Fu et al., 2018), such as schizophrenia (Yang et al., 2019). Meanwhile, Granger causality analysis (GCA) is an fMRI-based directed connectivity analysis method to assess the causality between different brain regions, which can provide more information on connectivity analysis than unidirectional FC (Wei et al., 2020; Huang et al., 2021). To date, the dynamic characteristics of ALFF and GCA have rarely been investigated to monitor the effect of acupuncture in patients with migraine.

To prove the cumulative therapeutic effect of acupuncture and explore the central mechanism of continuous acupuncture treatment in patients with MWoA, we designed this prospective study. After obtaining the brain areas showing different dynamic ALFF between patients with MwoA and HCs, we observed the changes in dynamic ALFF in these brain areas during the different acupuncture intervention periods. Then, the seed-based dynamic GCA analysis was used to understand the causality among these brain areas, which would help ensure the critical brain areas when the new alternative therapy was developed.

## Materials and methods

### Participants

Forty patients with MwoA were enrolled from outpatient in the Department of Neurology or Acupuncture at Shuguang Hospital affiliated to the Shanghai University of Traditional Chinese Medicine. Thirty-six healthy controls (HCs), who were aged, education level matched with the patients, and right-handed, were recruited. These volunteers had never been diagnosed with head trauma, alcohol/drug abuse, and neurological or psychiatric disorders. All participants signed written informed consent. This trial was approved by the Ethics Committee of the Shuguang Hospital affiliated to Shanghai University of Traditional Chinese Medicine and was registered on www.chictr.org.cn (ChiCTR1900023105). The diagnosis of MWoA was established according to the International Classification of Headache Disorders, 3rd Edition ICHD-III criteria (Olesen et al., 2018). Inclusion criteria included that all patients: (1) were 18–65 years old and right-handed; (2) had two to eight times of migraine attacks during the past month; (3) had at least 6 months of migraine history; and (4) had no physical therapy and prophylactic headache medications during the past month, and had no psychoactive or vasoactive agents during the last 3 months. The exclusion criteria included the following: (1) suffered from other types of primary or secondary headache; (2) had a history of a brain tumor or head trauma; (3) had any other neurological or psychiatric disorder; (4) were pregnant or breast-feeding; (5) had the contraindications for MRI or acupuncture.

### Study design

**FIGURE 1:**
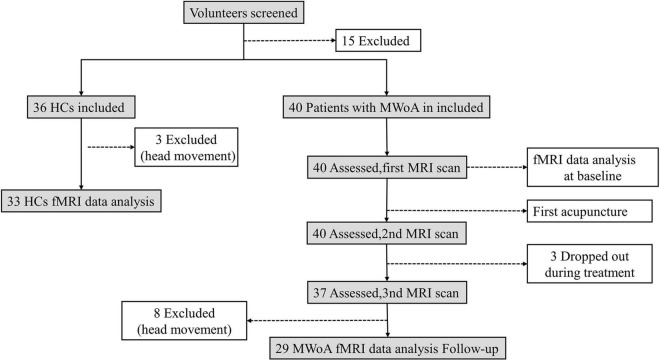
Flowchart displaying the screening, enrollment, and treatment. All abbreviations are defined in the Abbreviations section.

The total observation period for patients with MwoA in this study was 10 weeks. Weeks 1–4 were served as a baseline phase, and all patients recorded headache diaries during this phase. Weeks 5–10 were served as the intervention phase. During this phase, patients with MwoA were performed standard acupuncture treatment. All the patients kept recording their headache diaries. FMRI scans were administered before the first acupuncture session and immediately after the first and 12th acupuncture sessions for patients with MwoA (all fMRI scans were performed within 1 h before or after acupuncture). All patients with MwoA had been migraine-free for at least 72 h at the fMRI scans. The HCs group only received an fMRI scan at the baseline (Figure 1).

### Acupuncture treatment

**FIGURE 2:**
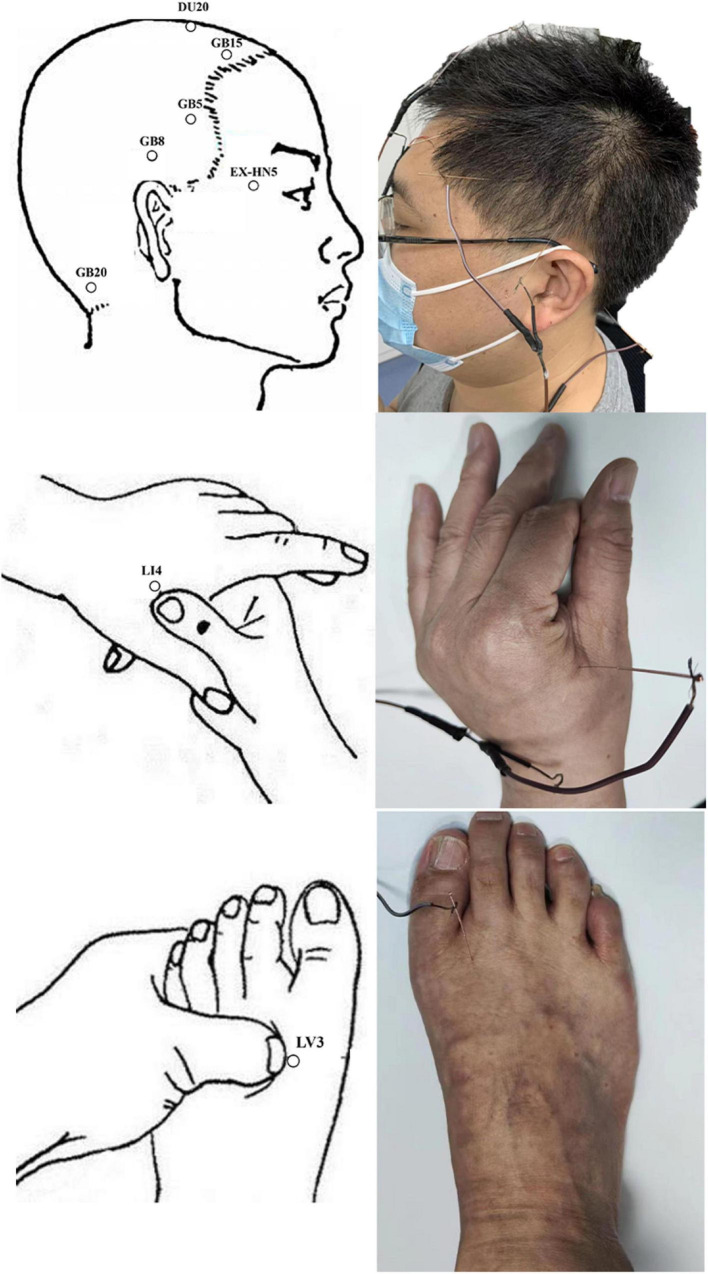
Diagram of electro-acupuncture treatment.

In our study, patients with MwoA were performed 12 sessions of acupuncture (twice a week, finished in 6 weeks), and every session lasted for 20 min. Acupoints were selected according to the standardized acupuncture protocol: Baihui (DU20), Taiyang (EX-HN5), bilateral Fengchi (GB20), Shuaigu (GB8), Xuanlu (GB5), Toulinqi (GB15), Hegu (LI4), and Taichong (LR3) (Chen et al., 2013; Zhao et al., 2017) (Figure 2). Two licensed acupuncturists (Wang B and Liu S, with 20 and 5 years of acupuncture experience, respectively) were responsible for all the acupuncture interventions. Sterile disposable acupuncture needles of 25–40 mm in length and 0.25 mm in diameter were inserted to achieve the sensation of deqi. Electrical stimulation was applied bilaterally at GB20 and GB8 at a frequency of 2 Hz and intensity ranging from 0.1 to 1.0 mA until the patient felt bearable. All participants agreed not to take any conventional medication for migraine during the study period. In case of severe pain, ibuprofen (as 300 mg extended-release capsules) was allowed as a rescue medication.

### Clinical assessments

During the 6 weeks from the first fMRI scan to finishing all the acupuncture sessions, the frequency of migraine attacks (days/month), visual analog scale (VAS) (0–10 scale, 10 being the most intense imaginable pain), the Self-Rating Anxiety Scale (SAS), the Self-Rating Depression Scale (SDS), and the Migraine-Specific Quality of Life Questionnaire scores (MSQ) were assessed. Adverse events associated with acupuncture, including bleeding, subcutaneous bleeding, severe pain, fainting, and local infection, were recorded at each treatment.

### Data acquisition

The MR scans were acquired on a 3T MRI scanner (uMR780 Platform, United Imaging Medical Systems, Shanghai, China) with a 12-channel flexible head coil at the MRI Center of Shuguang Hospital. The rest fMRI was obtained axially by a multislice gradient-echo echo-planar imaging (EPI) sequence, and the parameters were as follows: repetition time (TR) = 2,000 ms, echo time (TE) = 30 ms, flip angle = 90°, field of view = 240 mm × 240 mm, matrix = 64 × 64, 33 contiguous slices with 3.5 mm slice thickness, 240 time points. Structural images were acquired by a three-dimensional turbo fast echo (3D-TFE) sequence with a voxel size of 1 mm^3^, and the parameters were as follows: TR = 7.2 ms, TE = 3.1 ms, slice thickness = 1.0 mm, flip angle = 10°, field of view = 256 mm × 256 mm, matrix = 256 × 256, 176 slices without an interslice gap. A cushion was placed into the coil to fix the head and reduce motion. The participants were instructed to keep still with eyes closed, relax but not fall asleep, and try not to think about anything.

### Data preprocessing

The FMRI data preprocessing was performed by the DPABI software^1^ in MATLAB. The preprocessing course consisted of the following steps: (1) the images of the first 10 time points were discarded, and the images of the remained 230 time points were used for data analysis; (2) slice timing correction; (3) head motion correction (the translation or rotation motion in any given data did not exceed 2.0 mm or 2.0°); (4) the co-registered functional images were spatially normalized to the Montreal Neurological Institute (MNI) space and resampled to 3-mm cubic voxels; (5) linear trend removal was performed to reduce the effect of low-frequency drifts; (6) nuisance covariates regression (the white matter signal, the cerebrospinal fluid signal, and 24 head motion parameters); (7) lintemporal band-pass filtering at a frequency band of 0.01–0.08 Hz. After the head motion control, 11 subjects (three HCs and eight patients with MwoA) were excluded.

### Dynamic amplitude of low-frequency fluctuation analysis

The dynamic ALFF for each participant was performed by the DynamicBC (v2.2^2^) toolbox. Specifically, a temporal rectangular window was first chosen. Then, the ALFF values in each window were calculated. Window length was an essential parameter in resting-state dynamics computation. The “rule of thumb” in sliding-window length was that the minimum window length should be no less than 1/fmin (fmin = 0.01 Hz). Here, a window length of 50 TRs was considered as the optimal parameter to maintain the balance between capturing a rapidly shifting dynamic relationship and obtaining reliable estimates of the correlations between regions (Li et al., 2019; Cui et al., 2020). The sliding window was systematically shifted with a step size of five TRs (10 s) to calculate the dynamic amplitude of low-frequency fluctuation (dALFF) of each participant. The preprocessed data of each individual were segmented into 37 windows, and the ALFF map was obtained for each sliding window. Subsequently, we measured the variance of these maps by the standard deviation (SD) and evaluated the temporal variability of dALFF across 37 windows. The dALFF variability of each voxel was further transformed into a *z*-score by subtracting the mean and being divided by the SD of global values. Finally, the mean normalized dALFF maps were spatially smoothed using an isotropic Gaussian kernel of 8 mm full-width at half-maximum.

### Dynamic effective connectivity analysis

In this study, we performed seed-based dynamic Granger causality analysis (GCA) by the DynamicBC toolbox to detect the dynamic effective connectivity (DEC). The time series of each : region of interest (ROI) based on dALFF results was defined as the seed time series *X*, and the time course of voxels within the whole brain was defined as *Y*. A bivariate coefficient GCA to investigate the Granger causal influence between the per ROI and every voxel of the whole brain. A positive coefficient indicates that the activity in region *X* exerts a positive influence on the activity in region *Y*, whereas a negative coefficient indicated that the activity of region *X* exerted a negative effect on the activity of region *Y*. The dynamic GCA was estimated using the sliding window approach mentioned above, and the time series of each participant were also divided into 37 windows. Thus, for each participant, the averaged time course of the GCA coefficient of each ROI was extracted across 37 windows and concatenated to form a 2 × *W* × *N* matrix (where *W* denotes the number of windows and *N* denotes the number of ROIs). The DEC variability for each ROI was assessed with the SD of the averaged time course of the GCA coefficient across 37 windows. Finally, the dynamic GCA coefficient maps for all subjects were then converted to *z*-scores by Fisher *z*-transformation.

### Statistical analyses

Demographic characteristics were evaluated between patients with MwoA and HCs. The differences between the two groups in age and education level were analyzed with a two-sample *t*-test; χ^2^ test was used to analyze the difference in gender between the two groups. Two-sample *t*-tests or Mann–Whitney *U* test was used to compare the differences in the clinical variables between the two time points. *P* < 0.05 existed statistical difference.

Two-sample *t*-tests were performed to compare the difference in the dALFF variability between patients with MwoA at baseline and HCs within a gray matter mask with age, gender, education level, and head motion as covariates. The resultant T-maps were corrected for multiple comparisons correction by the Gaussian random field (GRF) theory (voxel *p* < 0.001, cluster *p* < 0.05, two-tailed).

To find the different effects of acupuncture during the different periods of treatment, we first performed repeated-measures one-way ANOVA to investigate the dALFF variability among the different periods. The SD value of each brain region with a significant difference between groups was extracted for statistical analysis in the SPSS version 25.0 (SPSS, Inc., Chicago, IL, United States), and *post hoc t*-tests were performed to detect the differences in dALFF variability between two periods (corrected by false discovery rate, *P* < 0.05).

For group-level analyses on DEC of the ROIs, the SD values of Zx → y and Zy → x dynamic GCA coefficient maps were calculated for each group. These maps were entered into repeated-measures one-way ANOVA to determine the difference among the different periods with age, sex, and education level included as covariates. Multiple comparison correction was performed based on Gaussian random field theory (GRF, voxel wise *p* < 0.001, cluster-wise *p* < 0.05, two-tailed). *Post hoc t*-tests were performed to detect the differences in DEC variability between two periods (false discovery rate corrected, *P* < 0.05).

Finally, the SD value of the dALFF and DEC variability in regions with significant differences in each individual with MwoA was extracted. Based on these regions, the Pearson/Spearman correlation analysis was used to probe the correlation of alterations in dALFF and DEC variability to the clinical data of patients with MwoA. The significance was set at a threshold of *p* < 0.05.

### Validation analyses

To validate the main findings of dALFF variability and DEC variability obtained from the sliding-window length of 50 TRs, we carried out auxiliary analyses with different sliding window lengths (30 and 80 TRs).

## Results

### Demographic and clinical characteristics at baseline

The demographic information and clinical characteristics of all the participants were presented in Table 1. There was no statistical difference in age (*p* = 0.408), education level (*p* = 0.313), and gender (*p* = 0.490) between patients with MwoA and HCs. The disease duration of the MwoA patients group was 16.21 ± 12.56 years, the frequency of migraine attacks was 5.14 ± 1.53 days, and the VAS score was 7.81 ± 1.39 (Table 1).

**TABLE 1:** Demographic and clinical characteristics of patients with migraine without aura and healthy controls at baseline.

Characteristics	Migraine patients at baseline (*n* = 40)	HCs (*n* = 33)	*P*-value
Age (years)	38.02 ± 9.79	33.26 ± 5.76	0.408
Gender (male/female)	6/34	7/26	0.490
Education (years)	15.02 ± 3.19	15.76 ± 1.76	0.313
Height (cm)	163.03 ± 6.05	165.12 ± 8.11	0.494
Weight (kg)	51.23 ± 6.23	53.89 ± 7.79	0.362
Disease duration (years)	16.21 ± 12.56	–	–
Frequency of migraine attack (days)	5.14 ± 1.53	–	–
VAS score	7.81 ± 1.39	–	–

### Differences in dynamic amplitude of low-frequency fluctuation analysis between patients with migraine without aura at baseline and healthy controls

The brain areas with a statistical difference in dynamic ALFF variability are shown in Table 2. Compared with HCs, patients with MwoA at baseline showed decreased dALFF variability in the RVM, superior lobe of left cerebellum (Cerebellum_Crus1_L), right inferior frontal gyrus, triangular part (IFGtriang. R), right median cingulate, paracingulate gyri (DCG.R), right precuneus (PCUN.R), left Inferior parietal, supramarginal, and angular gyri (IPL.L). Conversely, patients with MwoA showed increased dALFF variability only in the left Inferior occipital gyrus (IOG.L) compared with HCs.

**TABLE 2:** Brain regions with increased and decreased dALFF variability in migraine compared with healthy controls at the baseline.

Contrast	Brain regions	Peak MNI (*x*-, *y*-, *z*-)			Voxels	*T* score
MwoA < HCs	RVM	0	−28	−46	45	–3.77
	Cerebellum_ Crus1_L	−30	−69	−30	32	–3.39
	IFGtriang.R	42	21	15	39	–4.67
	DCG.R	6	12	36	21	–5.16
	PCUN.R	9	−57	54	26	–4.80
	IPL.L	−36	−63	54	37	–4.45
MwoA > HCs	IOG.L	−51	−78	−6	46	3.29

Gaussian Random Field theory correction, voxel P-value < 0.001, cluster P-value < 0.05. All abbreviations are defined in the Abbreviations section.

### Clinical outcomes

After 12 sessions of acupuncture treatment, the frequency of migraine attacks and the VAS score were significantly lower than those at baseline (*p* < 0.001). The SAS scores, SDS scores, and MSQ scores (restrictive, preventive, and emotional functional subscales) were significantly improved (*p* < 0.001) (Table 3). There was no adverse event associated with acupuncture.

**TABLE 3:** Clinical outcomes in patients with migraine without aura during the study period (*n* = 29).

Assessment points (weeks)	Frequency of migraine attack (days)	VAS score	SAS score	SDS score	MSQ score (restrictive subscale)	MSQ score (preventive subscale)	MSQ score (emotional subscale)	*P*-value
At baseline	5.14 ± 1.53	7.81 ± 1.39	46.04 ± 7.03	49.68 ± 8.29	58.24 ± 15.65	60.89 ± 21.27	63.46 ± 23.05	*P* < 0.001
At Treatment	1.85 ± 1.32	3.67 ± 1.20	38.41 ± 8.03	39.38 ± 9.87	80.33 ± 10.36	85.39 ± 13.22	87.67 ± 16.37	

All abbreviations are defined in the Abbreviations section.

### Dynamic amplitude of low-frequency fluctuation analysis during the different periods of treatment in patients with migraine without aura

**FIGURE 3:**
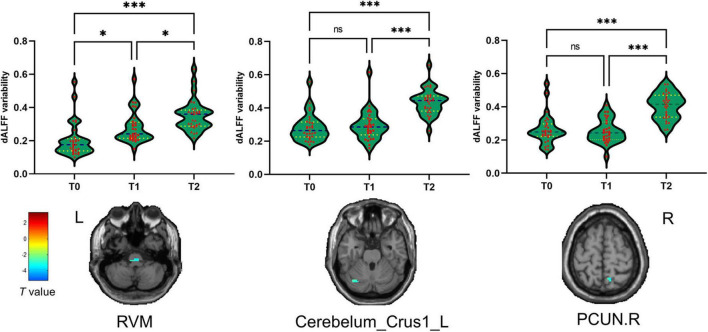
The dALFF variability significantly during the different periods of treatment. *p < 0.05, ^***^p < 0.001. The cool color represents decreased dALFF variability values. T0, the time point at baseline; T1, the time point after the first acupuncture session; T2, the time point after the 12th acupuncture sessions. All abbreviations are defined in the Abbreviations section.

The seven brain regions with a significant statistical difference in dynamic ALFF analysis above were extracted as the ROIs. According to the repeated-measures one-way ANOVA test, we found that the dALFF variability was significantly different in RVM, Cerebellum_Crus1_L, and PCUN.R. *Post hoc* tests revealed that the dALFF variability of patients with MwoA was significantly increased in the RVM after the first acupuncture session compared with that at the baseline, after all sessions of acupuncture compared with that at the baseline, and that after the first acupuncture session. The dALFF variability of the Cerebellum_Crus1_L and PCUN.R after all acupuncture sessions was significantly increased compared with that after the first acupuncture session. There were no statistical differences in the dALFF variability within the other ROIs (Figure 3).

### Seed-based dynamic Granger causality analysis analysis during the different periods of treatment in patients with migraine without aura

**FIGURE 4:**
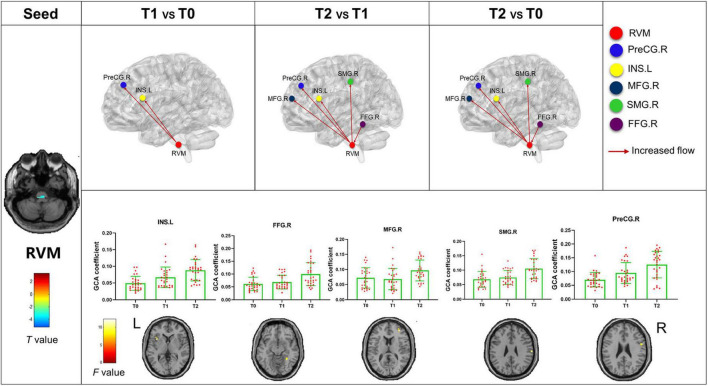
Abnormal effective connectivity pathways associated with the RVM. Each bar chart reflects the values of the dynamic GCA coefficient in the corresponding group. Fuchsia arrows indicate significantly enhanced DEC variability. T0, the time point at baseline; T1, the time point after the first acupuncture session; T2, the time point after the 12th acupuncture sessions. All abbreviations are defined in the Abbreviations section.

**FIGURE 5:**
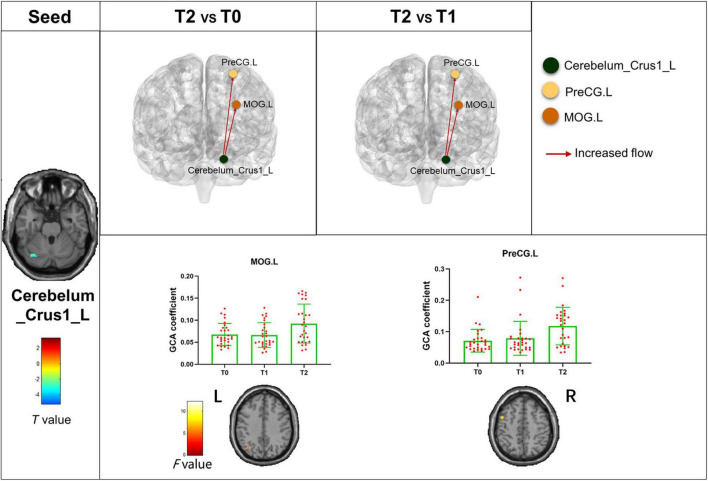
Abnormal effective connectivity pathways associated with the Cerebellum_Crus1_L. Each bar chart reflects the values of the dynamic GCA coefficient in the corresponding group. Fuchsia arrows indicate significantly enhanced DEC variability. T0, the time point at baseline; T1, the time point after the first acupuncture session; T2, the time point after the 12th acupuncture sessions. All abbreviations are defined in the Abbreviations section.

**FIGURE 6:**
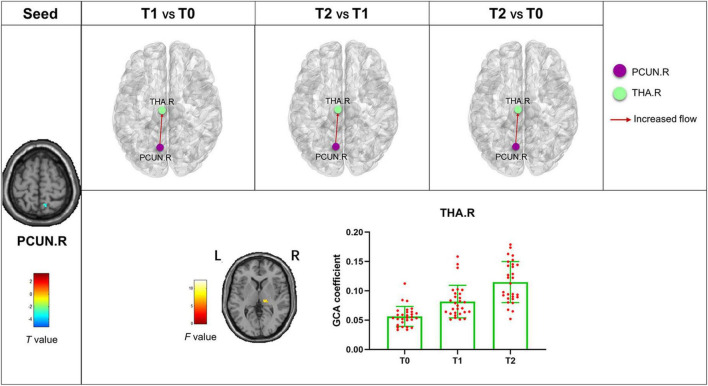
Abnormal effective connectivity pathways associated with the PCUN.R. Each bar chart reflects the values of the dynamic GCA coefficient in the corresponding group. Fuchsia arrows indicate significantly enhanced DEC variability. T0, the time point at baseline; T1, the time point after the first acupuncture session; T2, the time point after the 12th acupuncture sessions. All abbreviations are defined in the Abbreviations section.

The bivariate RVM-to-whole-brain dynamic GCA showed that the DEC variability from RVM outflow to the right middle frontal gyrus (MFG.R), the left insula (INS.L), the right precentral gyrus (PreCG.R), and the right supramarginal gyrus (SMG.R) were significantly enhanced in repeated-measures one-way ANOVA tests (Table 4). *Post hoc* tests revealed that the DEC variability from RVM outflow to INS.L and PreCG.R after the first acupuncture session were enhanced compared with that at baseline. The DEC variability from RVM outflow to MFG.R, INS.L, PreCG.R, and SMG.R was significantly enhanced after all acupuncture sessions compared with that at baseline and after the first acupuncture session. Next, whole-brain-to-RVM dynamic GCA showed that the DEC variability from the right Fusiform gyrus (FFG.R) inflow to RVM was significantly enhanced. *Post hoc* tests revealed that the DEC variability from FFG.R to RVM after all acupuncture sessions was significantly increased compared with that after the first acupuncture session (Figure 4). Furthermore, the bivariate Cerebellum_Crus1_L-to-whole-brain dynamic GCA showed that the DEC variability from Cerebellum_Crus1_L outflow to the left middle occipital gyrus (MOG.L) and the left Precentral gyrus (PreCG.L) (Table 4) was significantly enhanced. *Post hoc* tests indicated that the DEC variability after all acupuncture sessions significantly enhanced compared with that at the baseline and after the first acupuncture session, while there was no significant difference in the DEC variability after the first acupuncture session compared with that at the baseline (Figure 5). In addition, we also observed that the bivariate PCUN.R-to-whole-brain dynamic GCA displayed significantly enhanced DEC variability from PCUN.R outflow to the right thalamus (THA.R) (Table 4), and *post hoc* tests indicated that the DEC variability after the first acupuncture session was enhanced compared with that at the baseline, after all sessions of acupuncture compared with that at the baseline, and after the first acupuncture session (Figure 6). There was no statistical difference in DEC variability in the remained ROIs.

**TABLE 4:** The DEC variability significantly during the different periods of treatment.

Contrast	Brain region	Peak MNI (*x*-, *y*-, *z*-)			Voxels	*F* score
Causal outflow from RVM to the rest of the brain (*X* to *Y*)	INS.L	−36	12	3	16	6.67
	MFG.R	36	42	12	15	7.43
	SMG.R	57	−18	18	19	6.39
	PreCG.R	57	3	21	21	8.75
Causal inflow to RVM from the rest of the brain (*Y* to *X*)	FFG.R	24	−45	−18	23	6.54
Causal outflow from Cerebellum_Crus1_L to the rest of the brain (*X* to *Y*)	MOG.L	−33	−66	30	16	6.89
	PreCG.L	−39	6	39	18	8.44
Causal outflow from Precuneus_R to the rest of the brain (*X* to *Y*)	THA.R	3	−18	0	19	8.91

Gaussian Random Field theory correction, voxel P-value < 0.001, cluster P-value < 0.05.

### Correlation between dynamic amplitude of low-frequency fluctuation variability, dynamic effective connectivity variability, and clinical variables at baseline and after all acupuncture sessions

The SD values of the dALFF variability in RVM in patients with MwoA at the baseline were significantly positively correlated with the frequency of migraine attacks and negatively correlated with the disease duration at baseline (*p* < 0.001, *r* = 0.597; *p* = 0.033, *r* = −0.338, respectively). The frequency of migraine attacks was also significantly positive correlated with the SD value of the dALFF variability in Cerebellum_Crus1_L at baseline (*p* = 0.049, *r* = 0.314) (Supplementary Figure 1). The SD value of the dynamic GCA coefficient between MFG.R, INS.L, PreCG.R, SMG.R, and FFG.R with RVM was significantly positively correlated with the MSQ score and negatively correlated with the frequency of migraine attacks and VAS scores (Supplementary Figure 2 and Table 5) after all the acupuncture sessions. In addition, there were significant negative correlations between the SD value of the dynamic GCA coefficient from Cerebellum_Crus1_L to MOG.L and PreCG.L with the VAS scores (*p* = 0.006, *r* = −0.360; *p* = 0.009, *r* = −0.340, respectively), and there were significantly negative correlations between the SD value of the dynamic GCA coefficient from Cerebellum_Crus1_L to PreCG.L and frequency of migraine attack (*p* = 0.002, *r* = −0.397) (Supplementary Figure 3). Moreover, the VAS score and frequency of migraine attack were shown significantly negative correlations with the SD value of the dynamic GCA coefficient from PCUN.R to THA.R (*p* < 0.001, *r* = −0.544; *p* < 0.001, *r* = −0.691, respectively), and they were significantly positive correlated with MSQ score (*p* < 0.001, *r* = −0.568; *p* < 0.001, *r* = −0.675; *p* < 0.001, *r* = −0.566, respectively) (Supplementary Figure 4). No other significant linear correlation was observed.

**TABLE 5:** Correlation between the values of the dynamic GCA coefficient between MFG.R, INS.L, PreCG.R, SMG.R, and FFG.R with RVM and clinical variables after all acupuncture sessions.

ROIs	Frequency of migraine attack (days) (*r, p-*value)	VAS score (*r, p-*value)	MSQ score (restrictive subscale) (*r, p-*value)	MSQ score (preventive subscale) (*r, p-*value)	MSQ score (emotional subscale) (*r, p-*value)
INS.L	−0.515, <0.001	−0.508, <0.001	0.424, <0.001	0.412, 0.001	0.453, <0.001
MFG.R	−0.387, 0.004	−0.398, 0.002	0.408, 0.002	0.394, 0.005	0.422, <0.001
PreCG.R	−0.406, 0.002	−0.544, <0.001	0.468, <0.001	0.530, <0.001	0.508, <0.001
SMG.R	−0.534, <0.001	−0.323, 0.013	0.455, <0.001	0.527, <0.001	0.359, 0.006
FFG.R	−0.470, <0.001	−0.384, 0.003	0.429, <0.001	0.405, 0.002	0.340, 0.009

All abbreviations are defined in the Abbreviations section.

### Validation results

To verify the stability of our main results, other window sizes were included, such as 30 TRs and 80 TRs. The dynamic ALFF analysis and DEC analysis using different sliding-window lengths supported our main results (Supplementary Figures 5–8).

## Discussion

In this study, we applied the dALFF analysis to assess the abnormal changes of variability in patients with MwoA. The changes in dALFF and GCA variability after acupuncture were found. These alterations were associated with the clinical variables. The results could be summarized as follows: (1) the dALFF variability of patients with MwoA mainly were decreased compared with HCs at baseline, including RVM, Cerebellum_Crus1_L, PCUN.R, etc. (2) The decreased dALFF variability of RVM, Cerebellum_Crus1_L, and PCUN.R was progressively recovered, and the DEC variability was gradually increased after acupuncture treatment. (3) The dALFF variability of these brain areas was positively correlated with the frequency of migraine attacks and negatively correlated with the disease duration at baseline; the dynamic GCA coefficients were positively correlated with MSQ scores and negatively correlated with the frequency of migraine attacks and VAS scores after acupuncture treatment. Overall, our findings proved that the cumulative therapeutic effects of acupuncture treatment in patients with MwoA mostly focused on the changes in the brain dynamic activity and effective connectivity in RVM, Cerebellum_Crus1_L, and PCUN.R.

At first, we found the decreased dALFF variability of RVM in patients with MwoA. The ALFF has been proven to be an effective and reliable parameter for evaluating local intrinsic brain activity (Zang et al., 2007). As an extensive index of ALFF, the dynamic ALFF analysis subdivided the whole time series into multiple slices and then calculated ALFF in each slice. RVM was a part of the brainstem and a critical region of the descending pain modulatory system (Kong et al., 2010). ON and OFF cells within the RVM were activated by the onset and offset of noxious stimulation (Fields, 2004). But in migraine, its role was controversial. It was reported that patients with MwoA showed increased ALFF in posterior insula and putamen/caudate and reduced ALFF in RVM/trigeminocervical complex (TCC) (Li et al., 2017b). In another study, migraineurs had a significantly increasing fractional amplitude of low-frequency fluctuation (fALFF) in bilateral ventral posteromedial (VPM) thalamus and brainstem encompassing RVM and TCC (Kim et al., 2021). The inconsistent results for RVM might be due to the different subtypes of migraine, different sample sizes, and methodological variability. In our study, the decreased dALFF variability of the RVM was progressively recovered, and this finding was concordant with many studies reporting that acupuncture treatment could normalize the impaired descending pain modulatory system in migraine (Li et al., 2016, 2017b). The DEC variability from RVM outflow to INS.L and PreCG.R after the first acupuncture session were enhanced compared with that at baseline, and the outflow to MFG.R, INS.L, PreCG.R, and SMG.R and inflow to RVM from FFG.R were enhanced after all acupuncture sessions compared with the first acupuncture session. According to these results, the treatment effect of acupuncture was gradually increased following the increase in intervention times. The insula was associated with the different aspects of sensorimotor and cognitive control of speech production (Schwedt et al., 2015; Battistella et al., 2018). The precentral gyrus was the traditional movement-related higher cortex and included the primary motor area, the somatomotor cortex, and the lateral premotor area (Meier et al., 2008; Arca et al., 2021). The prefrontal cortex was the central brain region for executive functions (Ueda et al., 2018; Ashina et al., 2021). The fusiform gyrus was responsible for processing color information, face and body recognition, and the emotional expression of facial stimuli (Parvizi et al., 2012; Bassez et al., 2020). So, the cumulative therapeutic effect of acupuncture was embodied in affecting the cognitive, sensorimotor, speech, and executive functions of patients with MwoA through the RVM. This study also showed that the dALFF variability in RVM was correlated with the frequency of migraine attack and disease duration at the baseline, and these DEC variabilities of the RVM-to-whole-brain were correlated with the frequency of migraine attack and MSQ scores after acupuncture, which suggested that these brain regions might be the therapeutic targets in MwoA.

Our results also showed decreased dALFF variability in the Cerebellum_Crus1_L, IFGtriang.R, DCG.R, PCUN.R, and IPL.L. These regions were parts of the default, executive control, and cerebellar networks (Kim et al., 2017; Tu et al., 2019). A recent study revealed the abnormal dynamic low-frequency oscillation in the default mode network, salience network, and executive control network in patients with migraine (Chen et al., 2021). Another study showed the decreased ALFF value in the bilateral cerebellum posterior lobe, left cerebellum anterior lobe, bilateral orbital cortex, right middle frontal gyrus, bilateral occipital lobe, right fusiform gyrus, and bilateral postcentral gyrus in patients with migraine (Wang et al., 2016). These studies were generally consistent with the results of this study.

In addition, the dALFF variability of the Cerebellum_Crus1_L and PCUN.R was also slowly enhanced after acupuncture. Many studies have demonstrated the preventive effect of acupuncture on migraine might be through modulating the visual network, default mode network, sensorimotor network, frontal lobe network, and descending pain modulation system (Zou et al., 2019; Tu et al., 2020; Tian et al., 2021a,b). These are similar to the results of this study. The dALFF variability in Cerebellum_Crus1_L was correlated with the frequency of migraine attacks, which supported this perspective. Furthermore, acupuncture was a long-term and cumulative process (Zhao et al., 2017). Most of the studies of fMRI focused on the immediate effect of acupuncture on migraine. The changes in the whole process of acupuncture treatment were unknown. Thus, in our current study, the changes in dALFF variability at three time points during acupuncture treatment in patients with MwoA were observed to clarify the cumulative therapeutic effect of acupuncture. Moreover, the study additionally showed the enhanced DEC variability outflow from Cerebellum_Crus1_L to the left middle occipital gyrus and the left precentral gyrus and outflow from PCUN.R to the right thalamus enhanced gradually after the process of acupuncture treatment. The middle occipital gyrus was a vital component of the visual network and was responsible for the afferent input, integration, and perception of visual information (Zhu et al., 2018). Previous studies demonstrated a transient pathologic state with atypical thalamo-cortical connectivity in migraineurs (Coppola et al., 2021). These results might be able to intuitively explain the cumulative therapeutic effect of acupuncture. It was also shown that the cumulative therapeutic effects of acupuncture in migraine could be regarded as the consequence of interactions between pain modulation and cortical networks, which might provide an objective imaging marker to monitor the treatment in migraine. Moreover, these DEC variabilities of Cerebellum_Crus1_L and PCUN.R to-whole-brain were correlated with the frequency of migraine attack and MSQ scores after acupuncture treatment, which suggested that these brain regions might be the target areas for acupuncture improving MwoA.

Migraine attacks are often accompanied by psychiatric symptoms, such as anxiety, depression, agitation, panic, bipolar disorder, and sleep disturbances, such as difficulty falling asleep, excessive dreaming, and difficulty maintaining sleep (Tfelt-Hansen and Koehler, 2011). Therefore, when studying the psychosomatic symptoms associated with migraine, the comprehensive assessment of anxiety, depression, and sleep quality should be taken as a whole (McDermott and Ebmeier, 2009). This is a more comprehensive picture of the co-morbidity of migraine-related psychiatric disorders. In this study, the SAS and SDS scores of patients with MwoA improved significantly after acupuncture treatment, indicating that acupuncture can improve patients’ anxiety and depression. However, in subsequent correlational analyses, improvement in these symptoms was not associated with dynamic alterations of intrinsic brain activity and effective connectivity. This may be due to the lack of comprehensiveness of the fMRI study indicators selected for this study.

Our study has several potential limitations. First, no sham acupuncture group was set up during the longitudinal follow-up of acupuncture treatment, making it impossible to evaluate the interaction between the groups and time and the placebo effect. However, all patients with MwoA performed a series of fMRI scans, which enabled us to get longitudinal evidence of the cumulative therapeutic effect of acupuncture. Second, the optimal sliding window length to obtain dynamic changes in brain activity remains unclear. We selected 50 TRs as the window length based on previous studies, validated our results by using different sliding window lengths, and demonstrated that our findings were stable and not influenced by this factor. Third, the small sample size might weaken the liability of our results, and they should be further proved by the study with a big sample size in the future. Fourth, multimodal brain imaging (i.e., diffusion tensor imaging) was helpful for observing the changes in anatomical connectivity in patients with MwoA after acupuncture treatment, and it could be employed in the future.

## Conclusion

This prospective longitudinal study showed that the cumulative therapeutic effect of acupuncture in patients with MwoA. It manifested as the abnormal brain activity in RVM, Cerebellum_Crus1_L and PCUN.R were progressively recovered, and the effective connectivity of these brain areas with cognitive, sensorimotor, speech, executive, and cerebellar networks was gradually enhanced to make up for these abnormal brain activity.

## Data availability statement

The original contributions presented in this study are included in the article/Supplementary Material, further inquiries can be directed to the corresponding authors.

## Ethics statement

The studies involving human participants were reviewed and approved by the Ethics Committee of the Shuguang Hospital Affiliated to Shanghai University of Traditional Chinese Medicine and was registered on www.chictr.org.cn (ChiCTR1900023105). The patients/participants provided their written informed consent to participate in this study.

## Author contributions

WT and SZ designed the study. YC, YK, SLL, SSL, BW, ZG, YH, and HW performed the experiments. YC analyzed the data and was a major contributor in writing the manuscript. All authors read and approved the final manuscript.

## Conflict of interest

The authors declare that the research was conducted in the absence of any commercial or financial relationships that could be construed as a potential conflict of interest.

## Publisher’s note

All claims expressed in this article are solely those of the authors and do not necessarily represent those of their affiliated organizations, or those of the publisher, the editors and the reviewers. Any product that may be evaluated in this article, or claim that may be made by its manufacturer, is not guaranteed or endorsed by the publisher.

## Footnotes

1
http://www.rfmri.org/

2
www.restfmri.net/forum/DynamicBC

## Abbreviations

MwoA: migraine without aura
dALFF: dynamic amplitude of low-frequency fluctuation
GCA: dynamic Granger causality analysis
DEC: dynamic effective connectivity
ReHo: regional homogeneity
RVM: rostral ventromedial medulla
Cerebellum_Crus1_L: superior lobe of left cerebellum
IFGtriang.R: right inferior frontal gyrus, triangular part
DCG.R: right median cingulate and paracingulate gyri
PCUN.R: right precuneus
IPL.L: left inferior parietal, supramarginal and angular gyri
IOG.L: left inferior occipital gyrus
MFG.R: right middle frontal gyrus
INS.L: left insula
PreCG.R: right precentral gyrus
SMG.R: right supramarginal gyrus
FFG.R: right fusiform gyrus
MOG.L: left middle occipital gyrus
PreCG.L: left precentral gyrus
THA.R: right thalamus
MSQ: Migraine-Specific Quality of Life Questionnaire
VAS: visual analog scale
SDS: Self-Rating Depression Scale
TCM: Traditional Chinese Medicine
FC: functional connectivity
fMRI: Functional Magnetic Resonance Imaging
ROIs: region of interests
SD: standard deviation; healthy controls (HCs)
GRF: Gaussian random field.
