# Supplementary material for: The cumulative therapeutic effect of acupuncture in patients with migraine without aura: Evidence from dynamic alterations of intrinsic brain activity and effective connectivity
Source: Front Neurosci. 2022 Jul 19;16:925698. doi: 10.3389/fnins.2022.925698 (PMC9344052; doi:10.3389/fnins.2022.925698)
Supplement: Supplementary file 1 [file Data_Sheet_1.docx]

**Supplementary materials**


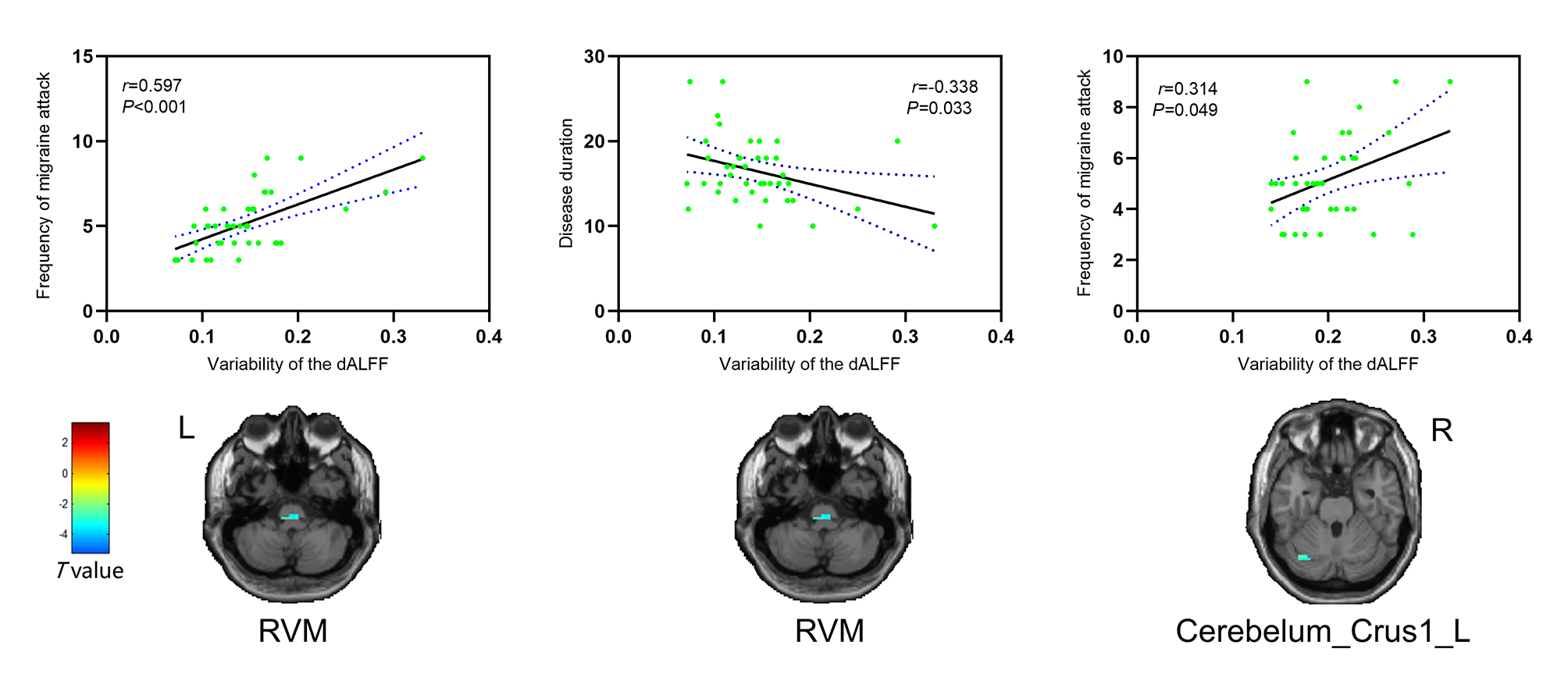


**Supplementary Figure 1.** The significant correlations between clinical variables and dALFF variability at baseline. All abbreviations are defined in the Abbreviations section.


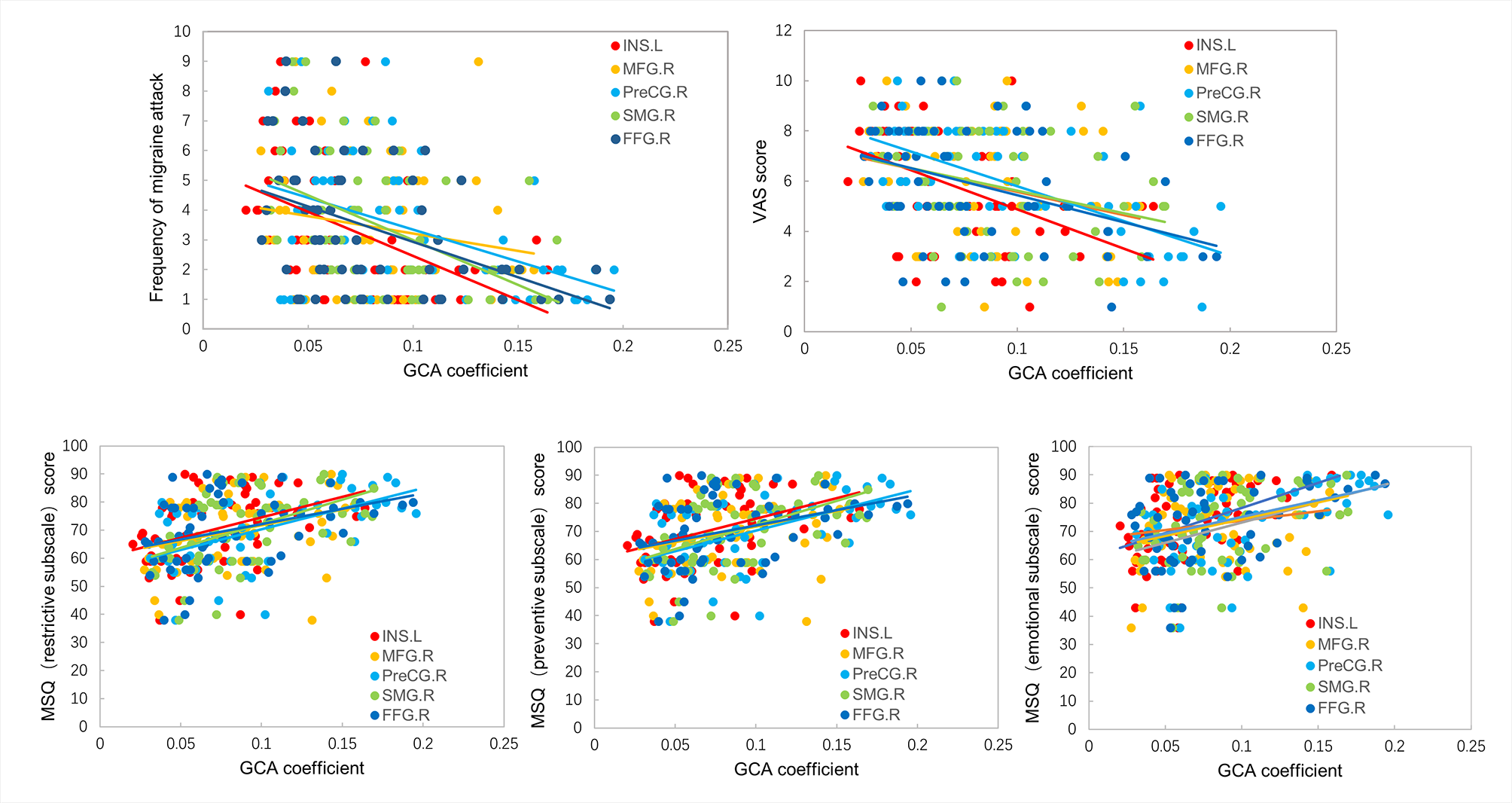


**Supplementary Figure 2.** The significant correlations between clinical variables and values of dynamic GCA coefficient from RVM to the rest of the brain during the different periods of treatment. All abbreviations are defined in the Abbreviations section.

**
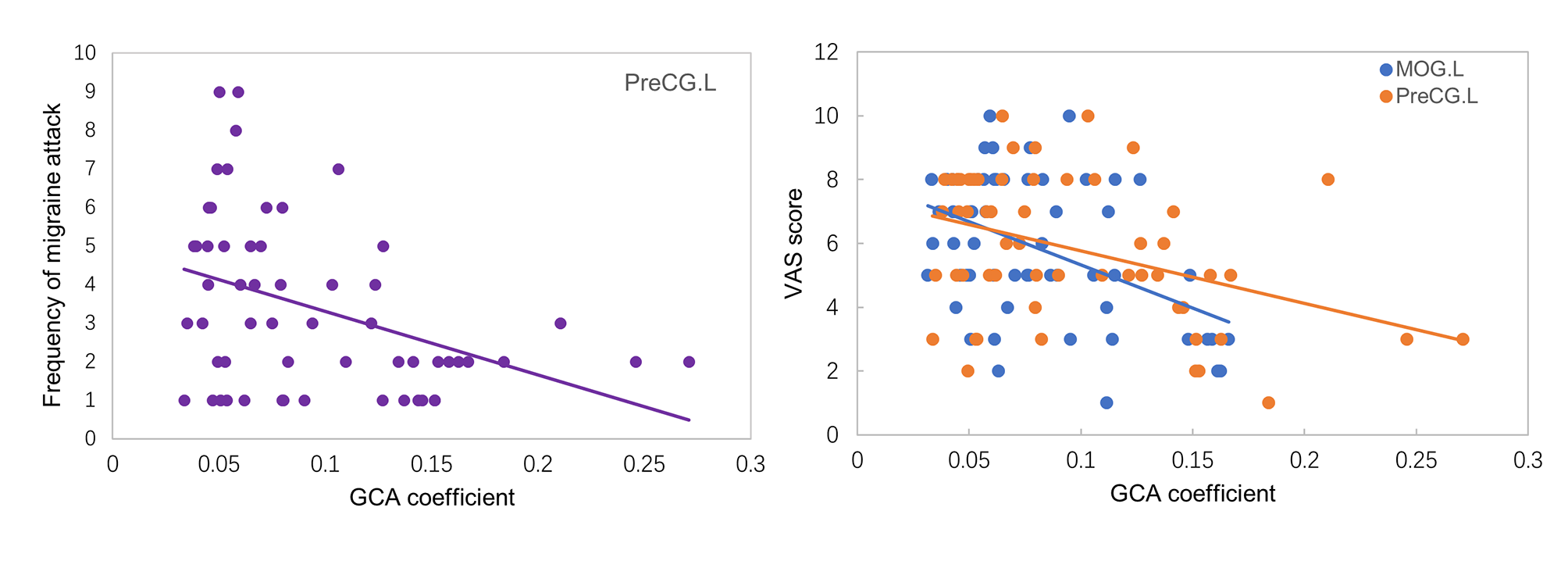
**

**Supplementary Figure 3.** The significant correlations between clinical variables and values of dynamic GCA coefficient from Cerebelum_Crus1_L to the rest of the brain during the different periods of treatment. All abbreviations are defined in the Abbreviations section.

**
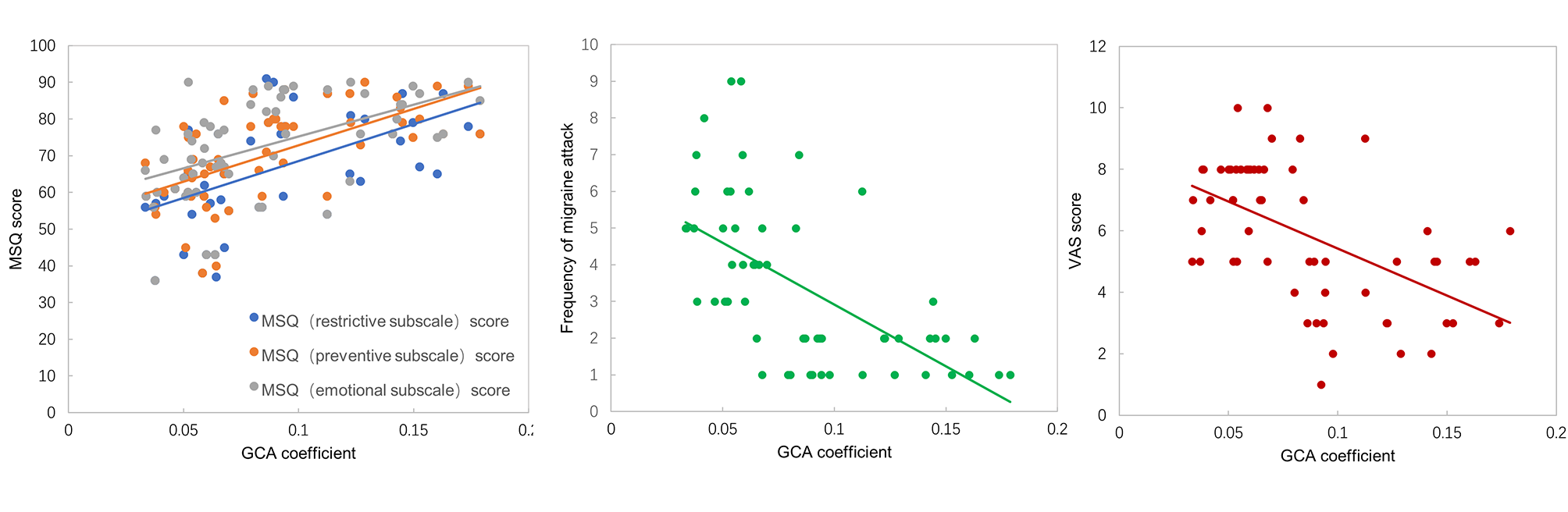
**

**Supplementary Figure 4.** The significant correlations between clinical variables and values of dynamic GCA coefficient from PCUN.R to the rest of the brain during the different periods of treatment. All abbreviations are defined in the Abbreviations section.

**
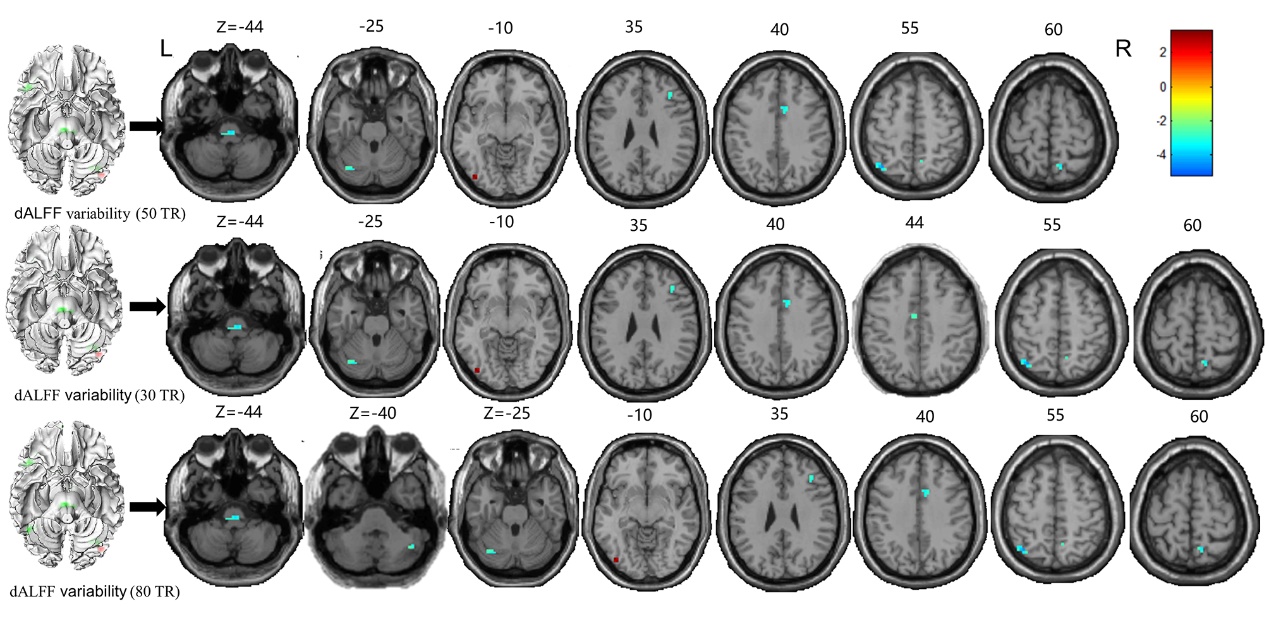
**

**Supplementary Figure 5.** Reproducibility results of the dALFF variability significantly at baseline in different sliding-window length. All abbreviations are defined in the Abbreviations section.

**
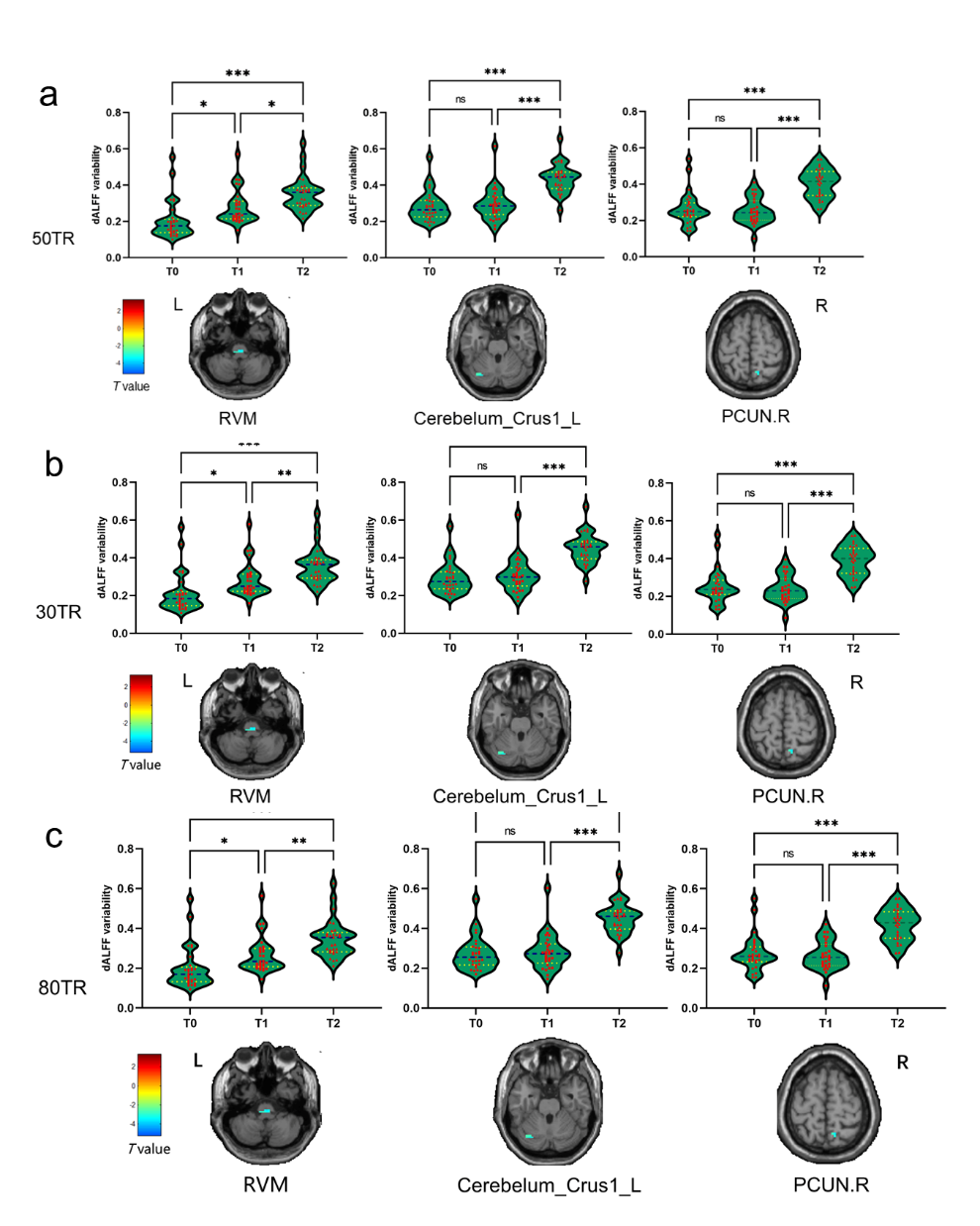
**

**Supplementary Figure 6.** Reproducibility results of the dALFF variability significantly during the different periods of treatment in different sliding-window length. T0: the time point at baseline; T1: the time point after the first acupuncture session; T2: the time point after the 12th acupuncture sessions. All abbreviations are defined in the Abbreviations section.

**
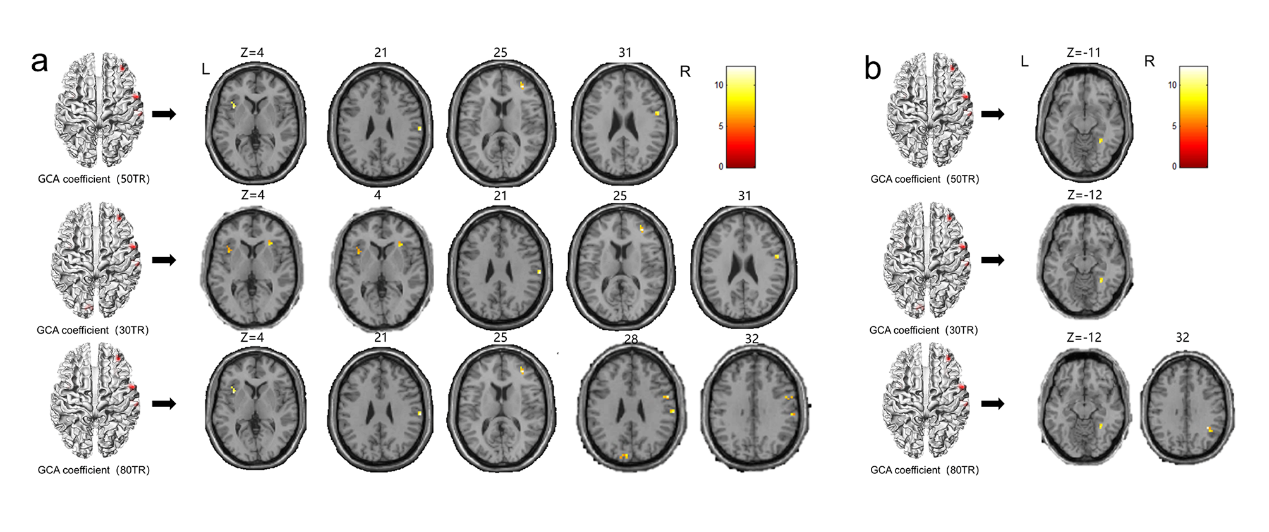
**

**Supplementary Figure 7.** Reproducibility results of abnormal effective connectivity from the RVM to other regions (a) and from the other brain regions to RVM (b) during the different periods of treatment in different sliding-window length. All abbreviations are defined in the Abbreviations section.

**
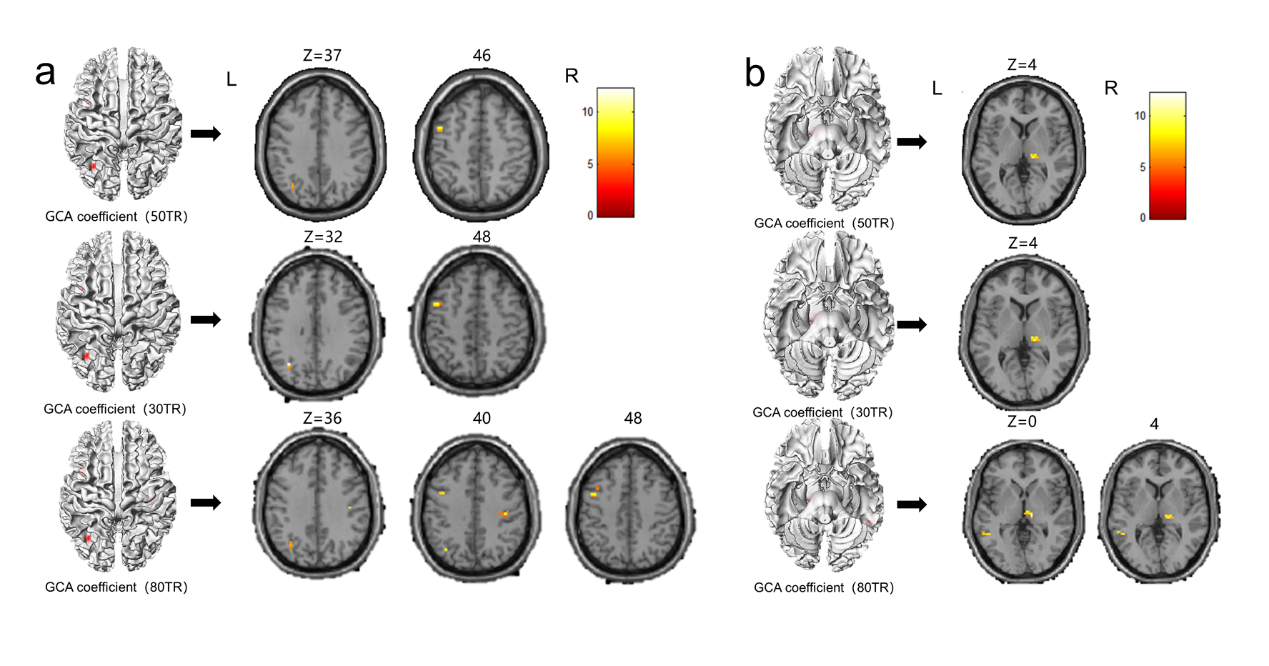
**

**Supplementary Figure 8.** Reproducibility results of abnormal effective connectivity pathways associated with the Cerebelum_Crus1_L (a) and PCUN.R (b) during the different periods of treatment in different sliding-window length. All abbreviations are defined in the Abbreviations section.
